# Self-management preferences in patients with mild cognitive impairment: A qualitative study

**DOI:** 10.3389/fpsyg.2022.955960

**Published:** 2022-10-14

**Authors:** Yuchen Jiao, Chang Liu, Jing Chang, Shiyu Zhou, Yan Ji

**Affiliations:** School of Nursing, Nanjing Medical University, Nanjing, China

**Keywords:** cognitive dysfunction, self-management, patient preference, qualitative research, China

## Abstract

**Purpose:**

Patients with mild cognitive impairment (MCI) require self-management, yet current self-management compliance is low. Taking patients’ preferences into account can improve the self-management situation. The purpose of this study is to look into MCI patients’ preferences for self-management in China.

**Methods:**

A qualitative research was conducted using semi-structured in-depth interviews with 21 patients recently diagnosed with MCI who were chosen by purposive sampling. These interviews were analyzed through thematic analysis and identified emerging themes.

**Results:**

Five themes of self-management preference were identified: (1) Preference for acquiring disease knowledge; (2) Preference for participating in drug therapy; (3) Preference for participating in exercise; (4) Preference for applying memory compensation strategy; (5) Preferences for emotional expression and response.

**Conclusion:**

Our study identified the specific preferences of MCI patients in China for the main self-management items. The findings are valuable insights for knowing MCI patients’ self-management content and preferences and provide better guidance for health practitioners to improve self-management compliance.

## Introduction

Mild cognitive impairment (MCI) is a preclinical stage of dementia and has a higher risk of progression to dementia than controls of the same age (Frederiksen et al., 2021). In China, the rising frequency of MCI is becoming a major public health concern, with approximately 15.54% of Chinese seniors over the age of 60 suffering from MCI (Jia et al., 2020). Despite the absence of a fatal hazard for a short time, the MCI state’s effects on an individual’s cognitive function, mood, physical function, and quality of life can result in a general decline in the patient’s physical and mental health, as well as a significantly increased risk of developing other diseases (Anderson, 2019). Prompt healthcare management can effectively slow or stop the progression in patients at this stage (Zou et al., 2019). Yet at the same time, MCI remains a chronic condition, it is critical to explore how to design a more sustainable management approach (Petersen et al., 2018).

The Journal of the American Medical Association (JAMA) has published an article describing the paradigm shift in chronic illness treatment during the 1980s—from conventional patient education centered on information transfer to fostering patients’ self-management. It places a premium on patient-centeredness, emphasizing the individual’s responsibility and ability to sustain health and cope with illness (Bodenheimer et al., 2002). Subsequently, the concept of patient-centered care was widely proposed. Scholl et al. sorted out the patient-centered principles, patient-centered activities, and enabling factors through a systematic review (Scholl et al., 2014). Studies have also shown that patient-centered care has a positive relationship with patient satisfaction, happiness, and other indicators (Rathert et al., 2013). Meanwhile, patient-centered self-management has been shown to boost patients’ awareness of their illness and their self-efficacy. It may help patients physically and psychologically and enhance their quality of life, clinical results, and access to health care (Quinn et al., 2016). With the advancement of cognitive impairment research, it has been shown that people with MCI may manage their health despite cognitive impairment (Lee et al., 2019).

However, individuals with mild cognitive impairment often have difficulty completing established self-management tasks. Patients are at risk of neglecting the importance of self-management due to attributing the disease to uncontrollable factors such as age or lacking confidence in their ability to manage (Santos et al., 2018; Cheng et al., 2021). In addition, even when patients complete self-management tasks during the intervention program, it is difficult for them to continue self-management without supervision from professionals or caregivers after finishing interventions. Tak et al. (2012) revealed that only less than one-fifth of the patients maintained compliance with the self-management after the tasks were completed. Based on the above, it is notable that MCI patients’ self-management situation is not optimistic.

Patients will be more motivated and benefit from self-management for longer if treatments are customized to their needs and preferences (Trappenburg et al., 2013). According to prior studies, when promoting self-management through peer support for patients with chronic diseases, it may only be beneficial if it is consistent with the personal preferences of the patient with chronic diseases, i.e., if the patient is willing to engage in peer support (Been-Dahmen et al., 2017; O'Connell et al., 2021). Evaluating patient self-management preferences also helps healthcare personnel give more individualized self-management support (SMS; O'Connell et al., 2021). While MCI patients’ cognitive function is moderately compromised, they can communicate their daily care preferences (Feinberg and Whitlatch, 2002). Some researchers believe that MCI patients’ self-management material and style should be tailored to their needs and preferences (Bensadon and Odenheimer, 2013). It could help patients gain self-management skills, better adapt to their needs, and receive tailored and participative counseling (Yu et al., 2017). Several studies have examined MCI patients’ preferences for medical care, focusing on treatment options and outcomes. Due to memory loss and lack of peers, MCI patients prefer choosing easy, safe, maneuverable, and group-cooperation treatments (Smith et al., 2018). For patients and their families, the most important outcome of care is quality of life, followed by self-control or coping skills for memory impairment (Chong et al., 2014). In addition to this, some patients with cognitive impairment exhibit more altruism, which may influence treatment decisions (Wehrmann et al., 2020). However, research on other self-management tasks and content is lacking.

There are few studies on MCI patients’ self-management preferences. The purpose of this study is to synthesize a list of self-management tasks for MCI patients through a literature review and to use this list as a framework for qualitative research to gain insight into patients’ thoughts and preferences for self-management content.

## Materials and methods

The report of the study findings followed the Consolidated Criteria for Reporting Qualitative Research (COREQ) checklist (Tong et al., 2007).

### Study design

This study is a descriptive study on the philosophical foundation of naturalistic inquiry (Athens, 2010), focusing on the preferences of self-management in patients with MCI. Based on the criteria for qualitative research, we conducted purposive sampling and used semi-structured interviews to gain data. Thematic analysis was used to produce the results. The study was approved by the Nanjing Medical University Ethics Committee (2021-No. 648) and was conducted following the Declaration of Helsinki.

Corbin and Strauss proposed a model of chronic disease self-management in a seminal report, stating that the core tasks of chronic disease self-management include three categories: medical management, role management, and emotion management (Corbin and Strauss, 1988; Begum et al., 2022). Our study will use this model as a basic framework and further complement the main self-management tasks under disease management, role management, and emotion management in MCI patients through a literature review.

With the themes “MCI,” “Self-Management,” “Medical Management,” “Role Management,” and “Emotional Management,” JYC and LC systematically retrieved the literature from PubMed from the establishment of the library until August 2021 to identify the primary self-management content or tasks of MCI patients. We restricted the language to English. JYC, LC, and CJ participated in the literature review and summary to make the list of MCI patient self-management tasks as clear as possible.

A systematic search and review of the literature revealed the following to be the common self-management tasks for MCI patients: The management of disease includes the acquisition of disease-specific knowledge, participation in drug therapy and drug trials, exercise, cognitive intervention, memory compensation measures, future planning, and the treatment of behavioral and psychological symptoms. Relationships with family and friends, daily activities, and social interaction are included in role management. Emotion management encompasses both emotional expression and emotional response. Details can be found in Table 1.

**Table 1:** A list of self-management tasks in patients with MCI.

**Dimension**	**Tasks**
Medical management	acquisition of disease-specific knowledge
	drug therapy
	drug trials
	exercise
	cognitive interventions
	memory compensation strategies
	future planning
	treatment of behavioral and psychological symptoms
Role management	relationships with family and friends
	daily activities
	social interaction
Emotional management	emotional expression
	emotional response

### Participants

The study was conducted in the neurology department of the Affiliated Brain Hospital of Nanjing Medical University, Jiangsu Province, China. The purposive sampling was used to recruit patients with rich potential information. We recruited people aged 45 or older because this is a high incidence population of MCI. Other inclusion criteria were as follows: (1) Patients with MCI (Group and Association, 2018): The diagnosis was made according to the criteria of mild cognitive impairment proposed in the 2018 Chinese Guidelines for the Diagnosis and Treatment of Dementia and Cognitive Impairment (5): Diagnosis and Treatment of Mild Cognitive Impairment published by the Writing Group of the Chinese Guidelines for the Diagnosis and Treatment of Dementia and Cognitive Impairment. The main diagnostic criteria: cognitive impairment reported by the patient or informant or observed by an experienced clinician; Objective evidence of impairment in one or more cognitive domains such as memory, executive function, language, visuospatial function (from neuropsychological testing); independent activities of daily living function; A diagnosis of dementia has not been reached. (2) Primary school education level or above. (3) The ability to communicate and hear is acceptable, and patients can cooperate with us during the interview. Exclusion criteria: (1) People with severe physical diseases or mental disorders. (2) Refusing to accept the interview or sign the informed consent. (3) Patients who are participating in other studies or drug trials. We cooperated with neurological physicians and neuropsychological measurement researchers to jointly identify patient eligibility criteria for MCI. Sampling continued until data saturation (*n* = 21) was reached (Saunders et al., 2018). In our study, we defined data saturation as thematic saturation. We conducted the qualitative interviews, followed by the interview transcription and data analysis immediately, and compared the results of the new interview with existing interviews. If the interview transcripts did not produce any new themes, we believed that we had reached thematic saturation (Constantinou et al., 2017). After reaching thematic saturation in our study, we continued to interview three patients with MCI, reconfirming that sufficient qualitative data had been collected to fully explain the specific content of self-management preferences of patients with MCI (Strauss and Corbin, 1990).

### Data collection

Firstly, two researchers (JYC, LC) contacted the participants and introduced background, purpose, and interview questions. Based on the voluntariness and confidentiality principle, we invited the patients to participate in our study and signed written informed consent. Then, the first authors (JYC, LC) conducted interviews. Researchers JYC and LC are graduate students who can understand local dialects, and have been in contact with MCI patients for a long time and have some experience in interviewing them. In addition, researcher JY has obtained a doctorate and has rich qualitative research experience to guide the interview. All the researchers have learned qualitative research courses systematically during the postgraduate period and mastered qualitative research methods and interview skills. The collection of qualitative data was completed from August to October 2021. Before the interview, the patients were required to cooperate with us to complete a short survey to collect primary demographic data. Then, the researcher reiterated the confidentiality of the study, obtained the patients’ oral informed consent and recorded it. We developed the interview outline based on the common self-management tasks for MCI patients (Supplementary material S1; Lorig and Holman, 2003). We first identified MCI patients’ willingness and perceptions of different self-management tasks of disease, since participation willingness is the basis of self-management preferences. Moreover, we conducted interviews around the list of self-management tasks to further analyzed and obtained specific self-management preferences of MCI patients. All interviews were conducted in a quiet and undisturbed office. During the whole interview, only the interviewer and patient were present. One-to-one interviews in Chinese were audiotaped and transcribed verbatim. During the interview, the researcher conducted interviews around the interview outline and briefly recorded the important statements mentioned by the patients. The researcher conducted more divergent questions according to patients’ actual conditions, such as conversation habits, and stimulated patients to express their self-management situations and preferences more clearly and richly through appropriate responses and guidance. After the interview, all audio and text materials were preserved properly, and two researchers transcribed word by word in Chinese within 24 h and marked them with codes to ensure anonymity. After the audio was transcribed, other researchers, including CJ and ZSY checked the transcription data against the interview audio to ensure the accuracy and completeness of the transcription. We conducted member-checking and provided the transcripts to the patients to verify the accuracy of the transcripts and ensure that the contents of the transcripts were consistent with the meanings expressed by the patients.

### Data analysis

We used thematic analysis (Castleberry and Nolen, 2018) to analyze the interview data and used triangulation to ensure the accuracy and credibility of the research findings. It includes five steps: (1) Two researchers (JYC, LC) read the interview transcripts continuously and were immersed in the original data to obtain a sense of integrity (Elo and Kyngäs, 2008). (2) After reading the transcripts more carefully, the researchers (JYC, LC) conducted open-coding for each interview transcript independently, split the data, and created meaningful groups, and then we discussed the coding results and agreed on the initial coding content. (3) We clustered and summarized by searching for similarities and differences and focused coding. Then, we clustered concepts around a similar phenomenon and put forward a more abstract concept, namely categorization. (4) We put the codes and categories into the context, enriched the connotation of categories, and finally improved the structure of categories to create the theme (Corbin and Strauss, 1990). (5) We reviewed whether the intercepted data was related to themes again and further retained, combined or discarded the candidate themes or sample interview data to obtain the most accurate expression (Braun and Clarke, 2006). At the end of the analysis, we discussed themes in the research group, and experts (JY) were invited to make suggestions on themes to improve the reliability of the data analysis. The data analysis reveals five themes covered in the next section. QSR-Nvivo12.0 software was used to store and manage all transcripts in this study.

## Results

We interviewed 21 participants, including 10 males and eight females. The age of patients ranged from 48 to 81 years, with an average age of 64. More than half of the participants (N = 18) had secondary school education, four patients were college or above, and three were primary education. Most of the participants (N = 17) lived in the city. Fourteen participants have retired and the remaining were at work. Participants were interviewed for an average of 37 min, with a duration of 24-78 min. We conducted neuropsychological testing on participants and mainly presented the scores of the Mini-Mental State Examination (MMSE; Folstein et al., 1975) and Montreal Cognitive Assessment Scales (MoCA; Nasreddine et al., 2005), which can represent the overall cognitive status of patients. It is particularly important for the diagnosis of MCI. In addition, the scores of the Activity of Daily Living Scale (ADL; Lawton and Brody, 1969) were also presented to assist diagnosis and understand the basic living conditions of the participants. Patients had an average MMSE score of 25 (range 21–30), MoCA had an average score of 18 (range 11–23), and ADL had an average score of 14 (range 14–15). The characteristics of the patients are shown in Table 2.

**Table 2:** Patients’ demographic characteristics.

**No.**	**Sex**	**Age**	**Education**	**Location**	**Occupation**	**Time (min)**	**MMSE score**	**MOCA score**	**ADL score**
1	Male	58	College	Urban	Employed	52	27	22	14
2	Male	67	Junior high school	Rural	Retired	34	27	15	14
3	Female	73	Junior high school	Urban	Retired	32	24	16	14
4	Female	52	Primary school	Urban	Employed	74	25	19	14
5	Male	48	Junior high school	Rural	Employed	39	29	22	14
6	Female	51	Junior high school	Urban	Employed	31	21	11	15
7	Female	72	High school	Urban	Retired	73	28	22	14
8	Male	53	High school	Urban	Employed	52	29	21	14
9	Male	74	Junior high school	Urban	Retired	30	23	16	14
10	Male	78	Junior high school	Urban	Retired	32	24	16	14
11	Female	68	Junior high school	Urban	Retired	31	22	16	14
12	Female	52	Junior high school	Urban	Retired	30	27	22	14
13	Male	81	College	Urban	Retired	32	24	20	14
14	Male	58	College	Urban	Retired	33	30	23	14
15	Female	73	High school	Urban	Retired	35	28	22	14
16	Male	72	High school	Urban	Retired	27	22	14	14
17	Male	58	College	Rural	Employed	78	26	18	14
18	Female	57	Primary school	Urban	Retired	25	22	16	14
19	Male	59	High school	Urban	Employed	30	22	14	14
20	Female	64	Junior high school	Urban	Retired	24	24	16	14
21	Female	67	Primary school	Rural	Retired	27	21	12	15

MMSE, Mini-mental state examination; MoCA, Montreal cognitive assessment.

During the analysis, five themes emerged: preference for acquiring disease knowledge, preference for participating in drug therapy, preference for participating in exercise, preference for applying memory compensation strategies, and preferences for emotional expression and response.

### Preference for acquiring disease knowledge: Access and content

Acquiring disease-related knowledge is a fundamental task for self-management in patients with MCI. We found that the preference of acquiring knowledge of disease in MCI patients is mainly reflected in the access and content of acquiring knowledge.

#### Access to disease knowledge

In the narratives of MCI patients who were willing to acquire disease knowledge actively, they preferred to obtain disease-related information by asking professional doctors, using online electronic devices such as television, computers, APP on the mobile phone (WeChat), and communicating with other patients or friends.

*The first point is consultation. I have some doctor friends and make a phone call at any time to ask about it. Furthermore, I would also search on WeChat or other websites. [Interviewee 1].*

*I did not know about it. I just went to the hospital and asked the doctor for professional advice. [Interviewee 17].*

*Most of the time, I learned about the memory problem on the Internet, such as by checking Baidu. [Interviewee 8].*

*These things (disease knowledge), I communicated with friends sometimes. Mrs. Shen (another patient in the ward) had some exchanges with me at the hospital, such as exercise. I will learn from her. Since I came here (hospital), I have learned many things. Mrs. Shen has a lot of vision and insight, and I learned a lot. [Interviewee 7].*

#### Content of disease knowledge

Compared with the cause of the disease, MCI patients preferred to understand the treatment plans, prognosis, and health guidance after discharge or at home. They were eager to know about effective disease treatment regimens and implement them as soon as possible to resume regular learning and life.

*I want to know how to treat it. If it cannot be cured, at least it can maintain or improve. If it becomes severe, it is very terrible. [Interviewee 3].*

*We also want to know if it will recover thoroughly in the future. How to cure it, and what can we do at home? [Interviewee 2].*

*It cannot solve the problem. Why do I know the cause of the disease? I want to see the treatment results, and I want to know how to cure it. [Interviewee 6].*

### Preference for participating in drug therapy: Regularity and personalization

Patients described their attitudes towards participating in medication. By analyzing patients’ interview data willing to participate in drug treatment, patients were more willing to take regular and long-term medication treatment to control disease progression. At the same time, patients with disease-related symptoms such as sleep disorders also had personalized medication needs.

#### Regularity

Long-term and regular medication is one of the preferences of participating in drug treatment in MCI patients. In China, most patients have a patterned presence in their minds: sickness - medical help-seeking - medication. They believe in the professionalism of doctors and recognize the importance and effectiveness of the medication.

*I want to take my medicine regularly, and I believe in the principle that professionals do professional things. Curing diseases still relies on doctors, and others are unreliable. [Interviewee 1].*

*Being sick, of course, needs to take medicine insistently. I listen to the doctor, and I fully trust my doctor. The doctor let me eat how long, how much, and I will follow the doctor’s advice. [Interviewee 16].*

#### Personalization

Patients had personalized medication needs for the accompanying symptoms or other accompanying diseases. They expected personalized guidance from doctors and hoped to improve cognitive impairment.

*My sleep status is not good now. I want to let the doctor prescribe a little personalized medicine to improve my sleep quality and mental state. Tell me to eat how much today, how much tomorrow. Is it suitable for my illness? [Interviewee 4].*

*I have high blood pressure, diabetes, and I eat medicine regularly. The doctor in the hospital prescribed this medicine. It is valuable for my memory to control these diseases well. [Interviewee 11].*

### Preference for participating in exercise: Group, convenience and safety

Exercise is an essential means of self-management in MCI patients. Most patients were willing to participate in sports and preferred group activities with low-to-medium intensity, convenient activities equipped with exercise equipment and suitable sports venues, and safe activities within the physical tolerance.

#### Group

Patients with MCI preferred group activities, such as square dancing, popular with middle-aged and older adults in China. They said such group activities were enjoyable and allowed them to interact with different individuals.

*I would like to participate in activities together. Single-person activities are diminutive. How does a person exercise? I also go walking with my companion. I participated in a square dance. If it does not rain, I dance every day. Recently, I stopped dancing for a while because of the epidemic. [Interviewee 7].*

*I would like to participate in group activities because I want to communicate with others. For example, dancing, playing cards, and singing are pleasurable activities for me. [Interviewee 4].*

#### Convenience

Patients with MCI preferred to use assistive devices for exercise, such as fitness equipment in the community, and exercise at convenient and suitable sports venues such as gyms, civic squares, and lakesides.

*There is a lake next to our neighborhood, and I always walk along the lake. [Interviewee 8].*

*We have a square. After getting up in the morning, I like to exercise in the fitness equipment. Then I will take a spin around our square, about one kilometer, usually for an hour or so. [Interviewee 2].*

*Exercise must be convenient, not too far from home. I need to take care of my grandson. If you have any fun activities in Nanjing, I certainly cannot come. It is too far. [Interviewee 18].*

*I do not have the energy now. Fortunately, the park is not far from my home. I can still go to the park every day. [Interviewee 20].*

#### Safety

Safe activities within the patients’ tolerance, such as walking near the residence, were essential for MCI patients.

*My legs are not good. I usually walk on the road for a while, close to home, to go back quickly. There are trees and stools over there. I am tired, and I can rest sometimes. There are fewer cars and people during the day. [Interviewee 12].*

*I am old. I have to take a walk next to my home. How dare I do any sports? I fell before, and my left hip was undergone surgery. [Interviewee 13].*

### Preference for applying memory compensation strategy: Self-helping and partial dependency

Patients with MCI may use memory compensation strategies spontaneously or be reminded by others to compensate for the negative impact of disease symptoms on life and work when the cognitive decline occurs early. Our study found that patients with MCI tended to use self-helping and partially item-dependent memory compensation strategies.

#### Type of self-helping

Some patients prioritized self-helping memory compensation strategies by forcing themselves to remember in self-learning methods (repeating, associative memory, pre-arranging). At the same time, such patients rejected the completely dependent memory compensation strategy that entirely relied on others’ help.

*I am going somewhere tomorrow, and I need to take something. Then I will take it at night before. I know that I will be busy in the morning and forget some things. My family would remind me too, but they do not know that I do not remember well. I still tend to do it by myself. [Interviewee 5].*

*I will force myself to write down some things I cannot remember, and it is exhausting.. [Interviewee 14].*

*I usually remember some situations deliberately, such as what I need to do and take. I will keep repeating. However, as soon as someone interrupts, I will forget all. [Interviewee 9].*

#### Type of partial dependency

Some patients preferred partial-item dependent memory compensation strategies, such as using external tools including alarm clocks, paper, monitoring, and mobile phone memos to cope with memory loss. This memory compensation strategy is widely used, and some patients (P1) call it “necessity” in life.

*I will take some measures. For example, sometimes I cannot remember something. I will use paper to copy it down. [Interviewee 10].*

*One method is using cell phone memos. I will check it one by one about something I need to take. It is hard to remember all things that I should take home. Besides, I usually write something in a notebook. After a while, I will comb these in my notebook, check what has not been completed, and re-listed them on the next page. It is a necessity for me in life. [Interviewee 1].*

*I installed a monitor on my doorstep to prevent forgetting closures or other situations. For example, I will open the monitor to see if the next door is closed when I go out. [Interviewee 4].*

### Preferences for emotional expression and response

Patients with MCI often had various negative emotions due to disease symptoms, adverse life events, changes in temperament. This study showed that most patients were willing to engage in negative emotional management, but they preferred to express these negative emotions naturally tolerated and venting. Then, they will fall back into another negative emotion of self-blame and sadness. Patients did not know how to manage negative emotions and were in helplessness.

#### Tolerance

Self-deliberately putting up with negative emotions is the most common way for MCI patients to deal with negative emotions. Negative emotions are difficult to adjust and correct in patients due to the lack of emotional expression.

*Although I am very angry, I am in control every time. After controlling myself, I am still irate, and I cannot vent. Bearing it is very uncomfortable. However, sometimes I said and vented, then I felt regretful to say that about my daughter. I felt like I had been unfriendly. [Interviewee 4].*

*When I am in a bad mood, I can only endure it. Sometimes I feel better, and sometimes I feel angrier and angrier. [Interviewee 21].*

#### Abreaction

Patients often experience out-of-control emotional conditions such as “losing their temper” to vent these negative emotions when faced with negative emotions. At the same time, they did not know how to adjust their emotional state to correct this emotional deviation.

*I want to control and be a little better, but I cannot control myself. I am bored, and my temper is hot sometimes. I would vent to my family, and I lost my temper. [Interviewee 5].*

*I used to have a good temper, but now if I am angry or in a bad mood, I especially want to throw things. [Interviewee 15].*

*How can I control it? Is taking this medicine able to control these negative emotions? [Interviewee 2].*

## Discussion

Our study investigated the self-management needs and preferences of patients with MCI based on the chronic disease self-management model. Through literature review, we extensively supplemented the specific self-management tasks of patients with MCI under medical management, role management, and emotional management in the model, to have a more comprehensive understanding of the scope of self-management tasks of patients with MCI. Subsequently, we developed the interview outline under the guidance of the chronic disease self-management model and common self-management tasks of MCI patients and carried out qualitative research, which was normative and guaranteed the reliability of the results. Finally, we found that MCI patients had a strong willingness to participate in the five self-management tasks, including acquiring disease knowledge, participating in drug therapy, exercising, applying memory compensation strategy, and emotional expression and response. These preferences for specific contents were characteristic and diverse.

Our qualitative findings indicated that MCI patients’ preferences for disease knowledge acquisition manifested themselves in two ways. In terms of disease knowledge, patients with MCI obtained it by consulting professional physicians, utilizing multiple network paths such as television, mobile phones, and computers, and communicating with other patients or friends. In terms of disease knowledge content, patients identified three critical components as treatment plans, prognosis, and home health guidance. MCI symptoms are often a sign of normal aging rather than a disease. Patients often choose to seek medical help to obtain relevant disease knowledge (Dai et al., 2013) when they have obvious and unexplained cognitive changes. We also found that utilizing multiple network paths is also an important way for MCI patients to acquire disease-related knowledge. With the intellectual development of modern society, conducting disease-related searching is becoming more popular in China. Despite their lack of technical knowledge, patients with MCI can increase their health knowledge if taught that the Internet can meet their disease management needs and given guidance and training (Klimova, 2017). However, given the complexity and variable quality of disease information available on the Internet, it is necessary to teach patients how to obtain scientifically accurate information about their disease. As a result, it is critical to provide consistent network resources and diverse information support to MCI patients during their health education. Furthermore, memory clinics for MCI patients, even in China, can only provide preliminary disease diagnosis and medication guidance, whereas patients prefer to learn about disease prognosis and treatment options (Palazzo et al., 2021). As Dai also pointed out (Dai et al., 2013), we discovered a gap between doctor-provided health services and patient preferences for disease knowledge. Patients’ capacity for self-management and subjective initiative is contingent upon their knowledge of the disease. Thus, when diagnosing and treating patients, healthcare workers must provide patients with sufficient disease information that corresponds to their preferences.

As a chronic disease, the primary treatment for MCI is always medications, no matter in the clinical application or the patients’ treatment preferences, which is a relatively common “clinical inertia” (Phillips et al., 2001). Currently, there is no proven, effective drug for MCI patients. Cholinesterase inhibitors are the commonly used drugs in clinical practice. However, there is no high-quality long-term research to prove its benefits in cognitive functions and it has apparent side effects. The American Academy of Neurology (AAN) recommends that these drugs not be used for MCI treatment (Petersen et al., 2018). There is a contradiction between patients’ high dependency on drugs and the lack of recommended medication for MCI. Meanwhile, our study only found that the MCI patients’ preference is long-term drug therapy but failed to find patients’ concrete preferences on drug dose, cost, effectiveness, safety and other aspects. This could be related to the patients in our study who were recently diagnosed with and were unfamiliar with the specific treatment drugs and regimens. The development of new drugs and drug trials are essential for cognitive impairment. However, our findings indicate that the majority of MCI patients in China are averse to participating in drug trials, even though we have informed these patients about the risks associated with drug trials. This will affect future drug research and development. On the one hand, they are worried about the security of the new drugs. On the other hand, the fact that MCI has no effect on a patient’s daily life significantly reduces the need for risky treatment. Thus, medication for MCI creates a vicious circle.

Exercise is an important protective factor and intervention mode for patients with MCI (Livingston et al., 2020). Guidelines and consensus in different countries emphasize the importance of exercise intervention (Petersen et al., 2018; Dunne et al., 2021; Frederiksen et al., 2021), and multiple clinical studies have confirmed the beneficial effect of exercise on cognition, body and emotion in patients with MCI (Ellis et al., 2019; Song and Yu, 2019). However, exercise interventions do have some drawbacks. For instance, exercise programs vary considerably, and patient compliance with exercise interventions varies significantly. As a result, it is critical to ascertain the exercise preferences of MCI patients. Our study discovered that one of the barriers to exercise for MCI patients is a lack of companions, and they preferred team sports, a finding that is consistent with Chong’s (Chong et al., 2014). Patients also have a need for exercise safety and prefer to engage in more secure activities. Health complaints have been identified as a significant barrier to participation in physical activities, particularly moderate to vigorous outdoor exercise, for patients with cognitive impairment (Chong et al., 2020). It corroborates our assertions. Additionally, given the decline in memory function in MCI patients, performing simple exercise patterns in an accessible environment is necessary, as complex exercise imposes greater cognitive demands (Chong et al., 2021). However, we discovered that some relatively young MCI patients preferred complex and professional activities such as table tennis and badminton. They believe that these activities can help the brain perform better in terms of reaction time and cognitive functions. As a result, it is necessary to tailor individualized exercise intervention programs to the unique characteristics and preferences of each patient.

Our study classified MCI patients’ memory compensation strategies as self-helping, partially dependent, or entirely dependent on people or objects due to memory loss. In our study, patients with mild cognitive impairment most frequently used self-helping and partial dependency compensation strategies to increase their independence and daily functional capacity (Tomaszewski Farias et al., 2018). In the Memory Compensation Questionnaire (MCQ), Dixon (Dixon et al., 2001; de Frias and Dixon, 2005) described five memory compensation strategies: internal aids (such as psychological imagination), spending more energy (such as focusing more on learning new names), spending more time completing tasks (such as slowing down reading), external aids (such as shopping lists), and recruiting others for help. This is consistent with our study’s classification, as the first three of the five categories above are consistent with a self-helping memory compensation strategy. A completely dependent memory compensation strategy refers to patients who entirely rely on others to remind and help them cope with the memory impairment. In our study, the majority of patients with MCI resisted using this strategy, which appears to be related to a fear of ridicule from others and the stigma associated with memory loss. However, older MCI patients (2 patients around 80 years old in our study) may be more inclined to employ an entirely dependent memory compensation strategy, with spousal reminders as the primary method. They were candid in describing how their spouse assisted them and reminded them of the important things in their lives, and how much they appreciated the spousal reminders.

Additionally, we discovered that patients with MCI would engage in any type of memory compensation strategy voluntarily. Numerous researchers (Frankenmolen et al., 2018; Tomaszewski Farias et al., 2018) have demonstrated that memory compensation training improves patients’ memory function and ability to function in daily life and that it can be used passively by researchers or healthcare workers to improve patients’ health outcomes. It necessitates further research into how to guide patients in properly training memory compensation strategies in order to assist them in coping with memory impairment and improving functions and health outcomes. Furthermore, we believe that memory compensation strategies should be approached dialectically. To prevent patients from becoming overly reliant on memory compensation strategies, caregivers should limit their use. This could result in delays in obtaining medical assistance and implementing other effective interventions.

Patients with MCI frequently struggle with emotional problems such as anxiety, depression, apathy, fear, and worry. In patients, negative emotions are linked to cognitive decline and poor physical health. Additionally, it may increase the likelihood of conversion to AD (Lu et al., 2009; Yates et al., 2017; Liew, 2020). As a result, addressing and resolving the emotional problems of MCI patients has become a critical and meaningful component of emotional management. In our study, MCI patients demonstrated a high level of willingness to deal with emotions and actively identify and manage emotional problems. Additionally, Smith et al. highlighted MCI patients’ expectations for improved emotional indicators (Smith et al., 2018). However, our research indicated that patients lacked knowledge of appropriate and effective emotional processing techniques, preferring to tolerate and vent emotional distress. We were unable to locate any previous studies examining emotion management preferences in MCI patients, but we did discover interventions that could help MCI patients with bad moods. Numerous people use methods based on various meditation practices to enhance their cognitive abilities and mood (Horrigan, 2007; Marciniak et al., 2020). A mindfulness-based stress reduction program (MBSR) had a positive effect on alleviating the depression of MCI patients and was widely accepted by participants (Marciniak et al., 2020). Additionally, studies have shown that increasing self-efficacy can help people with MCI feel better and have a higher quality of life (Tonga et al., 2020).

### Limitations and future research

There are some limitations to our study. First, the participants in this study were a small number of MCI patients from a specific region in China, and might not represent other regions’ patients with MCI, resulting in selection bias. However, thematic saturation was reached in our study, and characteristic information about MCI patients’ self-management preferences was fully obtained. Secondly, the interview data in our study are transcribed using Chinese and translated into English. The translation is done by one researcher, reviewed by another doctoral researcher, and any language issues were discussed and resolved by the research group. Although we have tried to avoid linguistic ambiguity, there might be expressions in native languages. In addition, patients in the study were recently diagnosed with MCI, and we did not consider longitudinal changes in self-management preferences. At the same time, we did not consider the impact of decreased executive function on daily living functions in MCI patients, so an assessment of this issue was lacking. We suggest that longitudinal studies of MCI patients’ preferences for self-management should be carried out to learn more about possible changes. Finally, given the inevitable limitations of the qualitative research perspective, we suggest that the quantitative study should be conducted for self-management preferences in MCI patients in the future.

### Practice implication

Our study identified the highest priority attributes and preferences for MCI patients’ self-management. It’s important to explore self-management preferences in patients with MCI because it prevents healthcare professionals from recommending or using undesired attribute guidance or requiring MCI patients to self-manage and can save scarce medical resources and protect patients from low-value interventions, increasing self-management compliance and improving patient-related health outcomes in turn (Hummel et al., 2012). In addition, the implementation of self-management is a multi-team effort in which healthcare professionals play an essential role in self-management support for MCI patients. Healthcare professionals should respect the self-management preferences of MCI patients and provide personalized guidance, treatment, care, and support based on patients’ preferences. More importantly, healthcare professionals should fully consider and respect MCI patients’ preferences within the scope of scientific and effective medical treatment. Excessive reliance on or compliance with patients’ preferences may influence the intervention effect. Healthcare professionals should balance patients’ preferences with scientific interventions to manage MCI more effectively.

## Conclusion

This study determined the specific contents of Chinese MCI patients’ preferences for self-management, including the preference for acquiring disease knowledge, participating in drug therapy, exercising, applying memory compensation strategy and emotional expression and response. We enlighten medical practitioners and emphasize the importance of patient preference on a scientific basis. This study is a preliminary attempt to explore the self-management preference of MCI patients, and it is necessary to accumulate and apply more findings to optimize the self-management process and improve the health outcomes of MCI patients.

## Data availability statement

The raw data supporting the conclusions of this article will be made available by the authors, without undue reservation.

## Ethics statement

The studies involving human participants were reviewed and approved by Nanjing Medical University Ethics Committee (2021-No. 648). The participants provided their written informed consent to participate in this study.

## Author contributions

YuJ and YaJ made contributions to conception and study design. YuJ and CL conducted the qualitative research and collected and analyzed qualitative data. YuJ wrote the first draft of the manuscript. YuJ, CL, JC, and SZ revised the manuscript. YaJ provided suggestions on writing and revised the article. All authors contributed to the article and approved the submitted version.

## Funding

This research was supported by National Natural Science Foundation of China (72204122) and the Connotation Construction Special nursing advantage discipline of Nanjing Medical University [szbf (2018) No.87].

## Conflict of interest

The authors declare that the research was conducted in the absence of any commercial or financial relationships that could be construed as a potential conflict of interest.

## Publisher’s note

All claims expressed in this article are solely those of the authors and do not necessarily represent those of their affiliated organizations, or those of the publisher, the editors and the reviewers. Any product that may be evaluated in this article, or claim that may be made by its manufacturer, is not guaranteed or endorsed by the publisher.
