# Supplementary material for: Self-management preferences in patients with mild cognitive impairment: A qualitative study
Source: Front Psychol. 2022 Oct 14;13:955960. doi: 10.3389/fpsyg.2022.955960 (PMC9614377; doi:10.3389/fpsyg.2022.955960)
Supplement: Supplementary file 1 [file Table_1.DOCX]

Supplementary Material

# S1: Qualitative Interview Outline

**Please tell me the information about the disease knowledge you obtained.**

1. Are you willing to take the initiative to acquire disease knowledge?
2. How do you prefer to obtain this knowledge? In what way?
3. What ki d of disease knowledge do you need most now, such as etiology, clinical manifestations, treatment, prognosis? Why is that?

# Next, I’m going to ask you about drug therapy and drug experimentation.

1. Do you think medicine is important for your disease or not?
2. Are you willing to take medicine for a long time? Why is that?
3. Will you forget to take the medication? In what ways would you remind yourself to take your medicine or remember to take it?
4. Are you willing to participate in drug trials? If you are not willing, what are your concerns? If so, what kind of help would you like us to provide?

# Please tell me your views on exercise.

1. Are you willing to carry out exercise intervention to manage the disease?
2. If you are willing, what kind of sports do you prefer? Can you elaborate on that? (Mode of exercise, duration, frequency, etc.)
3. If you are not willing, what do you think are the barriers?

# Please tell me your views and preferences on cognitive intervention.

1. Would you consider participating in such an intervention? Why is that?
2. What kind of cognitive intervention do you prefer?

# Please tell me about your use of memory compensation strategy.

1. Do you think memory compensation strategies are important or not in your life?
2. Will you use any compensation techniques in your life?
3. What type of compensation strategy do you like? Such as self-type, with the help of objects or reminders from others, etc.?

# Please tell me how you deal with the mental behavior symptoms? Such as anxiety, depression, irritability, etc.

1. For the moment, can you feel any changes or abnormalities in your behavior?
2. Did your family say anything to you?
3. Do you think the management of mental behavior symptoms is necessary or not?
4. How do you manage your psychological and behavioral symptoms?
5. What kind of help would you like?

# Could you tell me what you think about your plan for the future?

1. Are you willing to take the initiative to plan for the future?
2. In which aspects do you prefer to plan for the future?
3. What kind of help would you like?
4. Do you feel any obstacles?

# Please tell me about how you get along with your family members, friends.

1. Do you think it is necessary to establish and maintain good relationships with family and friends or not?
2. Has your relationship with your family or friends changed since your memory decline?
3. What troubles do you have when dealing with your relationships with them?
4. How have you dealt with these changes?

# Please tell me about your daily and social activities now.

1. Does your disease have any impact on your daily activities (such as doing housework, going out to buy groceries, taking care of yourself, etc.)? Does it have any impact on your social activities (going out to dinner, socializing with friends, managing money, working, etc.)?
2. Do these effects bother you? Do you feel the need to intervene?
3. What do you think needs to be done to deal with these impacts? Or how did you deal with it?

# Please tell me your current emotional state and information related to emotional coping.

1. Can you feel a change in your mood? What has changed?
2. Do you think emotional expression and management are important or not to your disease?
3. How do you prefer to express or deal with negative emotions related to disease? What kind of approach do you prefer?

# In addition to the above, what other self-management needs and preferences do you have? What other help would you like?

# S2: Interpretation of diagnostic criteria for patients with MCI

In the 2018 Guidelines for the Diagnosis and Treatment of Dementia and Cognitive Impairment in China (5) proposed by the Professional Committee on Cognitive Impairment Diseases of the Chinese Medical Doctor Association Neurology Branch, the main diagnostic criteria for MCI: cognitive impairment reported by the patient or informant or observed by an experienced clinician; Objective evidence of impairment in one or more cognitive domains such as memory, executive function, language, visuospatial function (from neuropsychological testing); independent activities of daily living function; A diagnosis of dementia has not been reached.

The standardized neuropsychological test scales we use include:

**①Mini-Mental State Examination (MMSE) and Montreal Cognitive Assessment Scales (MoCA):** used to measure the global cognition of patients, with scores for multiple cognitive domains such as orientation, immediate recall, delayed recall, attention, computational ability, language function and other measures of the sum of scores. The scoring standard varies according to the different versions of the scale and the educational level of the patients. The standard in this study is:

MMSE: primary school and below 20 points or more is normal; middle school and above 22 points or more is normal; college and above 23 is normal;

MoCA: primary school and below 19 is normal; middle school and above 22 is normal; college and above 24 is normal;

**②Auditory Verbal Learning Test (AVLT), Complex Figure Test (CFT):** used to evaluate the memory function of patients. AVLT can measure immediate recall and delayed recall, AVLT-delay < 5 points, AVLT-recognition < 20 points are abnormal. CFT is divided into two parts: imitation and recall of complex graphics. Generally, CFT imitation ≤ 33 points and CFT long-delay recall ≤ 12 points are considered abnormal.

**③ Digital Span Test (DST), Symbol Digit Substitution Modalities Test (SDMT):** mainly used to assess the patient’s attention.

DST includes Forward Digit Span Test (FDST) and Backward Digit Span Test (BDST), divided by education level, illiterate ≤ 5 points, primary school ≤ 6 points, middle school and above ≤ 7 into exceptions.

SDMT requires that according to the relationship between the specified symbols and numbers, fill in the corresponding symbols under the numbers, and fill in the number less than 34 within 90 seconds is abnormal.

**④ Trail Making Test (TMT):** It is mainly used to assess the executive function of patients. It consists of two parts (Trail-Making Test, parts A, TMT-A and Trail-Making Test, parts B, TMT-B). Among them, TMT-A mainly evaluates the patient's executive function and visual perception ability; TMT-B is mainly used to assess patients' mental flexibility. It is required to connect the numbers as required, and the completion time TMT-A > 70s and TMT-B > 180s are abnormal

**⑤ Verbal Fluency Test (VFT), Boston Naming Test (BNT):** used to assess the patient’s language function, VFT requires as many examples of a certain category as possible, VFT < 12, BNT < 21 was considered abnormal.

**⑥ Clock Drawing Test (CDT):** used to assess the patient’s visual space function. The CDT includes 14 specific scoring criteria, of which the first criterion reflects the clock drawing process, corresponding to CDT-A (Anchoring), with a total of 4 points; the remaining 13 items reflect the performance of the clock face, which is CDT-C (Clockface), a total of 26 points; A≤2 points, C≤17 points are abnormal.

⑦ In addition, we also used **Hasegawa Dementia Scale (HDS)** and **Clinical Dementia Rating (Clinical Dementia Rating, CDR)** to further evaluate the cognitive function of patients to exclude dementia. We also used the **Functional Activities Questionnaire (FAQ) and the Activity Of Daily Living Scale (ADL)** to assess the patients’ activities of daily living and social activities to assist in the diagnosis of MCI.
